# Switchable magnetic bulk photovoltaic effect in the two-dimensional magnet CrI_3_

**DOI:** 10.1038/s41467-019-11832-3

**Published:** 2019-08-22

**Authors:** Yang Zhang, Tobias Holder, Hiroaki Ishizuka, Fernando de Juan, Naoto Nagaosa, Claudia Felser, Binghai Yan

**Affiliations:** 1 Max Planck Institute for Chemical Physics of Solids, 01187 Dresden, Germany; 2 Leibniz Institute for Solid State and Materials Research, 01069 Dresden, Germany; 3 Department of Physics, Massachusetts Institute of Technology, Cambridge, MA 02139 USA; 4 Department of Condensed Matter Physics, Weizmann Institute of Science, Rehovot, 7610001 Israel; 5 Department of Applied Physics, University of Tokyo, Tokyo, 113-8656 Japan; 6 Donostia International Physics Center, P. Manuel de Lardizabal 4, 20018 Donostia-San Sebastian, Spain; 7 IKERBASQUE, Basque Foundation for Science, Maria Diaz de Haro 3, 48013 Bilbao, Spain; 8 RIKEN Center for Emergent Matter Science (CEMS), Wako, 351-0198 Japan; 9 Department of Applied Physics and Quantum Phase Electronics Center (QPEC), University of Tokyo, Tokyo, 113-8656 Japan

**Keywords:** Electronic properties and materials, Nonlinear optics

## Abstract

The bulk photovoltaic effect (BPVE) rectifies light into the dc current in a single-phase material and attracts the interest to design high-efficiency solar cells beyond the pn junction paradigm. Because it is a hot electron effect, the BPVE surpasses the thermodynamic Shockley–Queisser limit to generate above-band-gap photovoltage. While the guiding principle for BPVE materials is to break the crystal centrosymmetry, here we propose a magnetic photogalvanic effect (MPGE) that introduces the magnetism as a key ingredient and induces a giant BPVE. The MPGE emerges from the magnetism-induced asymmetry of the carrier velocity in the band structure. We demonstrate the MPGE in a layered magnetic insulator CrI_3_, with much larger photoconductivity than any previously reported results. The photocurrent can be reversed and switched by controllable magnetic transitions. Our work paves a pathway to search for magnetic photovoltaic materials and to design switchable devices combining magnetic, electronic, and optical functionalities.

## Abstract (web summary)

Two dimensional (2D) material with intriguing physical properties promises advanced electronic and spintronic technologies. Here the authors predict a magnetic photo-galvanic effect (MPGE) in bilayer 2D CrI_3_ due to the magnetism-induced asymmetry of the carrier velocity in the band-structure topology.

## Introduction

Under strong light irradiation, a homogeneous non-centrosymmetric material can rectify light into a dc current, called the bulk photovoltaic effect (BPVE)^1–7^. The induced open-circuit voltages can be much larger than the band gap. Thus, the BPVE displayed a promising potential in the solar energy conversion in ferroelectric perovskites^8–10^ and triggered the interest for the application of solar cells^11^ beyond the *p*–*n* junction design and more recently also the application of photodetectors^12^. It is believed that the shift current is dominant mechanism for the BPVE^2,4,13^. Shift current refers to real-space shift of conduction and valence Bloch electrons upon photoexcitation by a topological quantity, the Berry phase^14^. Therefore, ferroelectrics that exhibit intrinsic charge polarization are a promising direction in the search of BPVE materials. Furthermore, topological Weyl semimetals^15,16^ have recently been investigated for promising BPVE^17–23^, due to the large Berry phase in the band structure.

The essential requirement of the BPVE is inversion symmetry (${\cal{P}}$) breaking. Thus, the present guiding principle to design BPVE materials is to break the *crystal* centrosymmetry and sometimes to induce a strong charge polarization^24–27^. The lattice asymmetry or the polarization, however, is not the necessary condition for the inversion symmetry breaking. In this work, we show a large photogalvanic effect by a magnetic ordering which breaks *P* but preserves the parity-time symmetry (${\cal{P}}{\cal{T}}$, where ${\cal{T}}$ represents the time-reversal symmetry); therefore, no polarization exists. This phenomenon, called magnetic photogalvanic effect (MPGE), can generate a photocurrent even upon the linearly polarized light. But it cannot be described by the shift current that applies to non-magnetic systems. The MPGE is an intrinsic current response from the band-structure topology and distinct from the previously reported spin-galvanic effect^28^ and magneto-gyrotropic photogalvanic effects^29^ in semiconductor quantum wells (see ref. ^30^ for review), which are driven by an external magnetic field, and also the spin BPVE that generates a spin current^31,32^.

**Fig. 1:**
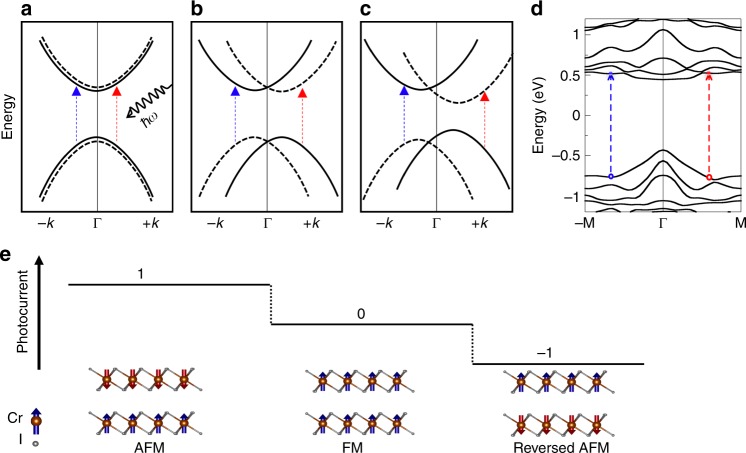
Band structure symmetry-breaking and magnetic structures of the bilayer CrI_3_. Schematics of band structures **a** with both inversion symmetry (${\cal{P}}$) and the time-reversal symmetry (${\cal{T}}$), **b** with only ${\cal{T}}$ but ${\cal{P}}$-breaking, and **c** with both ${\cal{P}}$- and ${\cal{T}}$-breaking. For both **a**, **b**, the light excitation (*ħω*) at +**k** and −**k** is symmetric to each other. However, such a symmetry is broken in **c**. As a consequence, excited electrons at +**k** and −**k** do not cancel each other in velocity, giving rise to a dc photocurrent. **d** The band structure of antiferromagnetic(AFM) bilayer CrI_3_. Here both ${\cal{P}}$- and ${\cal{T}}$ are broken as the case of (**c**), violating the **k** to −**k** symmetry. The spin-orbit coupling is included. The Fermi energy is shifted to zero. **e** The AFM, ferromagnetic (FM) and reversed AFM phases display three distinct responses to a linearly polarized light – positive current state(1), zero current state (0) and negative current state(−1)

The light excitation is known to generate an electron and hole pair in the solid. The velocity difference between the excited electron and hole may lead to a dc current. However, such a current usually vanishes because the velocity reverses its direction from **k** to −**k** in the momentum space. Such a symmetry in the band structure (see Fig. 1) is induced by either ${\cal{P}}$ or ${\cal{T}}$. So we can realize this photocurrent by breaking both ${\cal{P}}$ and ${\cal{T}}$ to avoid the velocity cancellation in the band structure, which is the core of the MPGE proposed.

We demonstrate the MPGE in a newly discovered two-dimensional ferromagnetic insulator, CrI_3_^33,34^. In the antiferromagnetic (AFM) phase of a CrI_3_ bilayer^33,35^, both ${\cal{P}}$ and ${\cal{T}}$ are broken while the combined symmetry ${\cal{P}}{\cal{T}}$ is preserved. We find that a giant dc photocurrent emerges in the visible-light window. Because the ${\cal{P}}{\cal{T}}$ symmetry forces the Berry phase to vanish, such a photocurrent is distinct from the shift current. The photoconductivity is sourced from the resonant optical transition in the asymmetric band structure. We found that the spin-orbit coupling (SOC) determines the extent of the momentum-inversion symmetry breaking and thus scales the amplitude of MPGE. The magnetic phase transition, which is controllable as demonstrated in recent experiments^35–39^, can be utilized to control the direction and amplitude of the induced current. When reversing all spins in the AFM phase, we can switch the current direction. When switching from the AFM phase to the ferromagnetic (FM) phase, we can completely turn off the current by recovering the spatial inversion. Therefore, we realize three states with different light-matter responses—positive, zero, and negative currents (as illustrated in Fig. 1e). Thus, the MPGE provides a new pathway to control the light-matter interaction by magnetism and to design optical storage/switch devices. Our findings can be generalized to multilayers, the bulk system, and other magnetic materials.

## Results

### Symmetry of 2D magnetic insulator CrI_3_

The recent discovery of 2D van der Waals magnetic insulators, such as CrI_3_, brings fascinating opportunities to design 2D magnetic devices. Bulk CrI_3_ is an FM insulator^34^. The FM coupling is preserved down to the monolayer limit^33,40^. In the bilayer and few-layer thickness, the interlayer coupling can be switched between AFM and FM by either an external magnetic field^37^ or electric gating^36,38^, giving rise to a giant magnetoresistance effect^35,39^. The atomic crystal of CrI_3_ exhibits inversion symmetry with two inversion centers, one inside the monolayer and the other in between neighboring monolayers. The AFM order reduces the crystal symmetry by removing the interlayer inversion center. Therefore, an AFM bilayer breaks the inversion symmetry, although an FM bilayer does not. Another important feature is that the AFM ordered phase preserves ${\cal{P}}{\cal{T}}$ symmetry while it breaks ${\cal{P}}$ and ${\cal{T}}$ independently; hence, no polarization exists in the ordered phase. Therefore, the bilayer CrI_3_ (see Fig. 1) is an ideal system to examine the photocurrent response, where the inversion symmetry is solely broken by the AFM order instead of the crystal structure. In the following, the ${\cal{P}}{\cal{T}}$ symmetry is found to be crucial to determine the mechanism of the photocurrent response.

We show the band structures of the bilayer calculated by the density-functional theory (DFT) including the spin-orbit coupling (SOC) in Fig. 1. All bands are doubly degenerate, which is protected by the ${\cal{P}}{\cal{T}}$ symmetry in the AFM phase. In contrast, such degeneracy is lifted in the FM phase (Supplementary Fig. 1). For the AFM phase, an important feature is the breaking of the **k** to −**k** symmetry. In a simple consequence, excited electrons (holes) at **k** and −**k** by the optical excitation (*ħω*) exhibit uncompensated velocities and lead to a nonzero dc current. In addition, the energy gap is slightly lower than the experimental value, which can be attributed to the known gap underestimation of DFT.

### Photocurrent of bilayer CrI_3_

In general, the photocurrent is a nonlinear effect. In this work, we provide a proper formalism (Equation (2)) to describe ${\cal{T}}$-breaking photocurrent by the second-order response theory (see Sec.2). We first evaluate the photocurrent response of the bilayer CrI_3_ using the general formalism based on Bloch wave functions of the realistic material. Then we prove that this formalism can be reduced to a simple form of the resonant optical transition (Equation (6)) in the presence of the ${\cal{P}}{\cal{T}}$ symmetry.

**Fig. 2:**
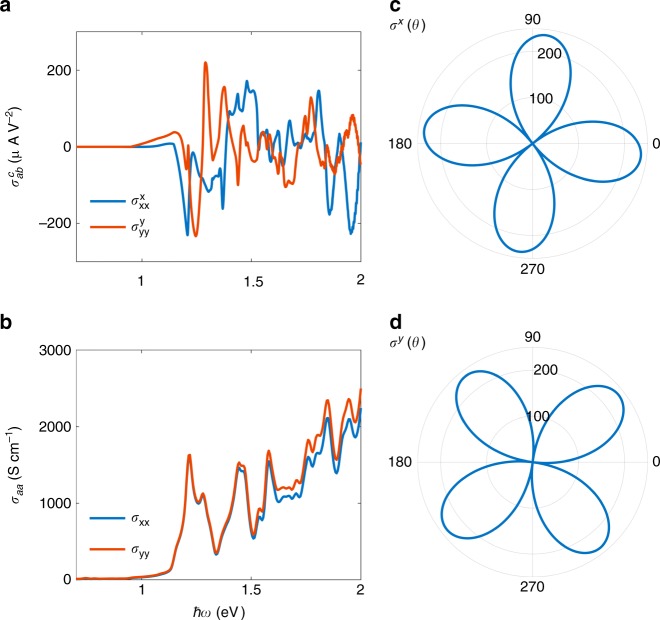
Calculated photoconductivity in response to the linearly polarized light. **a** The photon energy (*ħω*) dependence of $\sigma _{xx}^x$ and $\sigma _{yy}^y$. **b** The linear-response optical conductivity *σ*_*xx*_ and *σ*_*yy*_. **c**, **d** The angle dependent photoconductivity *σ*^*x*^(*θ*),*σ*^*y*^(*θ*) [cf. Eq. (3)] in the same unit as **a** for *ħω* = 1.2 eV and *ħ*/*τ* = 1 meV. *x* and *y* are the directions of the current and *θ* is the angle between the electric field of light and the *x*-axis

Under the irridation of the linearly polarized light, the FM bilayer exhibits vanishing photocurrent due to inversion symmetry. However, the AFM phase displays a large photocurrent conductivity (more than 200 *μAV*^−2^ for a relaxation time *τ* ≈ 0.4 ps, i.e., *ħ*/*τ* = 1 meV) in the visible-light range (see Fig. 2). This value is higher than that of many known BPVE materials reported so far^6,24,26,41–46^. We note that the value of the photocurrent is proportional to the relaxation time (see Supplementary Fig. 3). Here we choose the *τ* value according to the magnitude of experimental reports on other transition metal dichalcogenides^47,48^. We note that the photocurrent reverses its direction when reversing all spins in the AFM phase. Therefore, the FM, AFM, and reversed AFM represent three photocurrent states, 0 (no current), 1 (positive current), and −1 (negative current), respectively, as illustrated in Fig. 1. In addition, for circularly polarized light, we do not observe any photocurrent for both AFM and FM bilayers, which is constrained by the 2D point group symmetry.

## Discussion

We describe the BPVE response by the general second-order Kubo formalism^2^ and then drive the MPGE in the condition of ${\cal{T}}$-breaking. The general theory accounts for the steady-state short-circuit photocurrent with the relaxation time approximation. The conductivity $\sigma _{ab}^c$ (*a*, *b*, *c* = *x*, *y* in 2D) represents the photocurrent *J*^*c*^ generated by the dipole electrical field of light, **E** = (*E*_*a*_(*ω*), *E*_*b*_(*ω*), 0),

1 $$J^c = \mathop {\sum}\limits_{ab} {\sigma _{ab}^c} E_a^ \ast (\omega )E_b(\omega )$$

2 $$= \mathop {\sum}\limits_{ab} {\frac{{\pi e^3}}{{\omega ^2}}} Re\left\{ {\mathop {\sum}\limits_{l,m,n}^{\Omega = \pm \omega } {{\int}_{BZ} {{\textstyle{{d{\mathbf{k}}} \over {(2\pi )^d}}}} } f_{ln}{\textstyle{{v_{nl}^av_{lm}^bv_{mn}^c} \over {(E_{nm} - i\hbar /\tau )(E_{nl} - \hbar \Omega - i\hbar /\tau )}}}E_a^ \ast (\omega )E_b(\omega )} \right\},$$

where $v_{nl}^a = \left\langle {n,{\mathbf{k}}} \right|\hat v^a\left| {l,{\mathbf{k}}} \right\rangle$, $\hat v^a$ is the velocity operator, *τ* is the relaxation time, *d* is the system dimension, *f*_*ln*_ = *f*(*E*_*l*_) − *f*(*E*_*n*_), *f*(*E*_*n*_) is the Fermi-Dirac distribution, *E*_*nm*_ = *E*_*n*_ − *E*_*m*_, *E*_*n*_ ≡ *E*_*n*_(**k**), and |*n*, **k**〉 are energy and wave functions, respectively, at **k** for the *n*th band.

The photocurrent conductivity matrix $\sigma _{ab}^c$ is shaped by the symmetry of the AFM bilayer. The three-fold rotational symmetry and ${\cal{P}}{\cal{T}}$ lead to only six nonzero tensor elements, $\sigma _{xx}^x = - \sigma _{yy}^x = - \sigma _{xy}^y = - \sigma _{yx}^y$ and $\sigma _{yy}^y = - \sigma _{xx}^y = - \sigma _{yx}^x = - \sigma _{xy}^x$. Here only two of them (such as $\sigma _{xx}^x$ and $\sigma _{yy}^y$) are independent, since *x* and *y* directions are not equivalent in a hexagonal lattice. For a linearly polarized light **E** = (cos*θ*, sin*θ*, 0)*E*_0_cos(*ωt*), according to Eq. (1) the photocurrent along the *x* and *y* direction is

3 $$\begin{array}{l}J^x = \sigma ^x(\theta )E_0^2 = [\sigma _{xx}^x\cos (2\theta ) - \sigma _{yy}^y\sin (2\theta )]E_0^2\\ J^y = \sigma ^y(\theta )E_0^2 = [ - \sigma _{yy}^y\cos (2\theta ) - \sigma _{xx}^x\sin (2\theta )]E_0^2.\end{array}$$

The photocurrent is sensitive to the polarization direction *θ*, as we show in Fig. 2c, d. Following Eq. (3), if $\sigma _{yy}^y$ is zero, the maxima of *σ*^*x*^(*θ*) is located at *θ* = 0, 180°. Because $\sigma _{yy}^y$ is generically nonzero at a given frequency; however, the maxima of *σ*^*x*^ shift away from *θ* = 0, 180° in Fig. 2c. Such an anisotropy is usually measured to deduce the conductivity tensor elements in experiment.

In contrast, for the circularly polarized light, the photocurrent is forced to vanish by the 2D point group symmetry (*C*_3_) despite that it may appear in a 3D material. In other words, $J^x$ = $\mathop {\sum}\limits_{ab} {\sigma _{ab}^x} E_a^ \ast E_b$ = $\sigma _{xx}^xE_x^ \ast E_x + \sigma _{yy}^xE_y^ \ast E_y + \sigma _{xy}^xE_x^ \ast E_y + \sigma _{yx}^xE_y^ \ast E_x$ = $0$ for a circular polarized light with *E*_*y*_ = *iE*_*x*_. Such a dramatic distinction between light polarization provides a simple, useful hallmark to verify these results on 2D materials. We will focus our discussions mainly on the linearly polarized light in the following.

The general formalism (Equation second-kubo) can be simplified when special symmetries appear. The BPVE arises as a consequence of the three-band (*l*, *m*, *n*) interference in the optical excitation. (i) The response function vanishes to zero if the inversion ${\cal{P}}$ exists, because the numerator $N_{lmn}({\mathbf{k}}) = v_{nl}^av_{lm}^bv_{mn}^c$, the product of three matrix elements, is odd to **k**. (ii) If ${\cal{P}}$ is broken but ${\cal{T}}$ appears, *N*_*lmn*_(**k**) = −*N*_*lmn*_(−**k**)^*^. Then only the imaginary part of *N*_*lmn*_(**k**) contributes nonzero values to the photocurrent. Therefore, the linearly and circularly polarized lights are related to the imaginary and real parts of the energy denominator, respectively, in Eq. (2). For a ${\cal{T}}$-symmetric insulator, Eq. (2) can be simplified to the shift current and injection-current formalisms^2,4,49^ for the linearly and circularly polarized lights, respectively. In non-magnetic systems, this implies that the circular photocurrent scales linearly with the scattering rate, while the linear photocurrent is independent of the scattering rate. Here, the velocity matrix is commonly transformed to the length gauge^49^.

For the bilayer AFM CrI_3_ that respects ${\cal{P}}{\cal{T}}$ but breaks ${\cal{P}}$ and ${\cal{T}}$ independently, the response function exhibits a unique symmetry. Because ${\cal{P}}{\cal{T}}$ requires $N_{lmn}({\mathbf{k}}) = N_{l\prime m\prime n\prime }^ \ast ({\mathbf{k}})$, the numerator [$N_{lmn}({\mathbf{k}}) + N_{l\prime m\prime n\prime }^ \ast ({\mathbf{k}})$] in Eq. (2) is always real, where *l*′, *m*′, *n*′ are ${\cal{P}}{\cal{T}}$ partners (degenerate in energy) of *l*, *m*, *n*, respectively. Therefore, the linearly and circularly polarized lights are related to the real and imaginary parts of the energy denominator, respectively, opposite to the ${\cal{T}}$-symmetric case.

To establish the intuitive correlation between the band structure and the BPVE, we decompose Eq. (2) into two-band and three-band process^2,21^. The former corresponds to a direct resonant transition from |*l*〉 to |*n*〉, where *n* = *m*. By considering (*E*_*n*_ − *E*_*m*_ − *iħ*/*τ*)^−1^ = −*iτ*/*ħ* in Eq. (2), the response function can be derived,

4 $$\sigma _{ab}^c = - \frac{{\pi e^3\tau }}{{\omega ^2\hbar }}\mathop {\sum}\limits_{l,n;\Omega = \pm \!\omega } {{\int}_{\!\!\!\!BZ} {\frac{{d{\mathbf{k}}}}{{(2\pi )^d}}} } f_{ln}\frac{1}{2}\{ v_{nl}^a,v_{ln}^b\} v_{nn}^c\delta (E_{nl} - \hbar \Omega ),$$

where $\frac{1}{2}\{ v_{nl}^a,v_{ln}^b\} = \frac{1}{2}(v_{nl}^av_{ln}^b + v_{ln}^av_{nl}^b) \equiv Re(v_{nl}^av_{ln}^b)$ and the *δ*-function is derived from the imaginary part of (*E*_*nl*_ − *ħ*Ω − *iħ*/*τ*)^−1^. Such two-band photocurrent is proportional to the relaxation time *τ* and decided by the resonant transition according the selection rule *E*_*nl*_ = ±*ħω*. We further transform Eq. (4) to the length gauge,

5 $$\sigma _{ab}^c = \frac{{\pi e^3\tau }}{\hbar }\mathop {\sum}\limits_{l,n} {{\int}_{\!\!\!\!BZ} {\frac{{d{\mathbf{k}}}}{{(2\pi )^d}}} } f_{ln}\frac{1}{2}\{ r_{nl}^a,r_{ln}^b\} \Delta _{ln}^c\delta (E_{nl} - \hbar \omega ),$$

where $r_{nl}^a = i\left\langle n \right|\partial _{k_a}\left| l \right\rangle$ is the position matrix element and $v_{nl}^a = r_{nl}^aE_{nl}/\hbar \equiv r_{nl}^a\Omega$ if *E*_*nl*_ − *ħ*Ω = 0, and $\Delta _{ln}^c = v_{ll}^c - v_{nn}^c$. This formula is very similar to the the known ${\cal{T}}$-symmetric injection-current expression(Equation (56) in ref. ^4^), except that in ref. ^4^ the injection current is integrated over the imaginary part of the position matrix $r_{nl}^ar_{ln}^b$ due to ${\cal{T}}$. In contrast, Eq. (5) evaluates its real part due to ${\cal{P}}{\cal{T}}$. Suppose a linearly polarized light with the polarization along *x*, Eq. (5) looks more intuitive in the following form,

6 $$\sigma _{xx}^c = \frac{{\pi e^3\tau }}{\hbar }\mathop {\sum}\limits_{l,n} {{\int}_{\!\!\!\!BZ} {\frac{{d{\mathbf{k}}}}{{(2\pi )^d}}} } f_{ln}|r_{ln}^x|^2\Delta _{ln}^c\delta (E_{nl} - \hbar \omega ).$$

It represents an excitation from *l* to *n* with dipole transition rate $|r_{ln}^x|^2$. The excited electron (hole) with finite velocity $v_{nn}^c$ ($v_{ll}^c$) induces a dc current. If $v_{nn}^c$ at **k** and −**k** cancel each other exactly when ${\cal{P}}$ or ${\cal{T}}$ exists, the photocurrent from Eq. (5) or (6) vanishes. Only when both ${\cal{P}}$ and ${\cal{T}}$ are broken, a nonzero photocurrent may exist.

The three-band contribution refers to *n* ≠ *m*. We can exclude a trivial case that |*n*〉 and |*m*〉 are degenerate in energy for example protected by ${\cal{P}}{\cal{T}}$, because $v_{nm}^a = 0$ if so. When *E*_*n*_ ≠ *E*_*m*_, the real part of the denominator in Eq. (2) is *τ*-independent. Thus the three-band process generates a photocurrent that is robust against scattering, different from the two-band transition. The three-band process evaluate (*E*_*nm*_ − *iħ*/*τ*)^−1^ in the denominator. Thus, the ratio between the three-band and two-band contributions are (*ħ*/*τ*)/*E*_*nm*_ that is usually in the order of meV/eV. Then the three-band contribution is much smaller than the two-band one. In calculations, we indeed find that the photoconductivity is predominantly produced by the two-band transition (the three-band contribution is less than 0.5%, See the supplementary Fig. 2).

It is useful to find a simple indicator for the nonlinear photoconductivity. Because the photocurrent is proportional to the resonant transition rate, the imaginary dielectric function or the optical conductivity may provide such an indicator. In other words, the optical conductivity is the sum of all allowed transitions equally while the photoconductivity is to sum them with the velocity weight (see Eq. (6)). As shown in Fig. 2c, the optical conductivity *σ*_*xx*_ is strongly correlated to $\sigma _{xx}^x$. In addition, the photoconductivity indeed scales linearly to the relaxation time *τ* in our calculations (Supplementary Fig. 3).

**Fig. 3:**
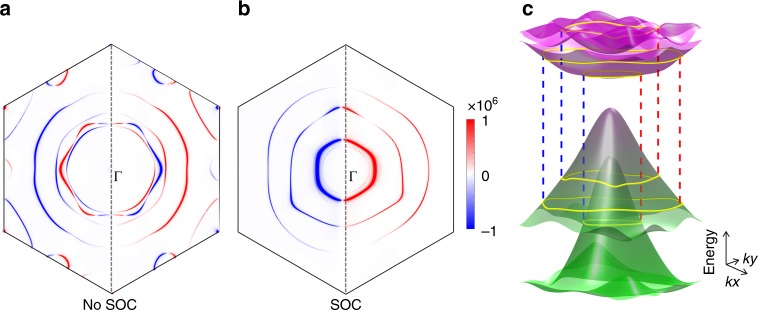
**a**, **b** Distribution of the photoconductivity ${\boldsymbol{\sigma }}_{{\boldsymbol{xx}}}^{\boldsymbol{x}}$ in the first Brillouin zone. Without including the spin-orbit coupling (SOC), the **k** to −**k** symmetry appears while finite SOC breaks such a symmetry. The ring-like shape indicates the resonant optical transition between valence (*E*_*l*_) and conduction (*E*_*n*_) bands by the selection rule *E*_*n*_ − *E*_*l*_ = *ħω* (1.2 eV). **c** Transitions from top two valence bands to the bottom two conduction bands. The yellow rings indicate the transition paths and correspond to the large-$\sigma _{xx}^x$-amplitude rings in (**b**)

Figure 3 shows the $\sigma _{xx}^x$ distribution in the momentum space for the AFM bilayer at *ħω* = 1.2 eV. It is clear that the photoconductivity is contributed by resonant transition channels between the valence and conduction bands. Because of the breaking of both ${\cal{P}}$ and ${\cal{T}}$, these channels are not symmetric between **k** and −**k** anymore. We point out that SOC play a significant role here despite that it is less obvious from Eq. (6). The strength of SOC represents the amount of the **k** to −**k** symmetry breaking in the band structure. When SOC is absent, the AFM band structure is still symmetric (see the supplementary Fig. 1). This is because of the spin rotation symmetry SU(2). Therefore, the net photocurrent vanishes, as shown in Fig. 3a. Finite SOC locks the the spin orientation with respect to the lattice and breaks the momentum-inversion symmetry (see Fig. 3b), resulting in a nonzero photocurrent. The SOC modifies the band structure by opening a gap at the band crossing points. So peaks of $\sigma _{xx}^x$ usually correspond to the transitions involving these anti-crossing gap regions.

In reality, the substrate or the gate may modify the bilayer electronic structure by breaking the crystal inversion between two layers. It is practical to investigate how robust the ${\cal{P}}{\cal{T}}$-symmetry-induced photocurrent behaves in the presence of such perturbation. We apply an out-of-plane electric field *E* to represent the perturbation. *E* induces a potential drop between two layers and breaks the ${\cal{P}}{\cal{T}}$ symmetry of the bilayer. We calculate the photoconductivity using Eq. (2), where both the real and imaginary parts of *N*_*lmn*_(**k**) contribute. The photoconductivity remains relatively robust even for a large *E* = 0.005 V/*a*.*u*. (1 *a*.*u*. = 0.53 Å) for the AFM bilayer (see the Supplementary Fig. 4). On the other hand, the FM phase starts to exhibit nonzero photocurrent when *E* breaks its inversion symmetry. At *E* = 0.005 V/*a*.*u*., the photoconductivity is of the same order as in the AFM case.

In addition, the MPGE can be easily generalized to multilayers and other magnetic materials (such as Cr_2_Ge_2_Te_6_^50^ and VSe_2_^51^). Some magnetic orders of a trilayer CrI_3_, for example, ↑↑↓ or ↑↓↓, can break the inversion symmetry and generate the MPGE. Different from a bilayer, ${\cal{P}}{\cal{T}}$ is broken here. Then the real and imaginary parts of *N*_*lmn*_(**k**) contribute to the photoconductivity in Eq. (2). Magnetic orders such as ↑↑↑ and ↑↓↑ preserve the inversion symmetry and produce no photocurrent. Further, hetero-structures of layered materials and twisted layers provide vast possibilities. Beyond 2D materials, the MPGE may also exist in 3D systems, specially AFM materials with the ${\cal{P}}{\cal{T}}$ symmetry and strong SOC, such as Cr_2_O_3_^52^, Mn_2_Au^53^, and CuMnAs^54,55^.

To summarize, we discover a magnetic photogalvanic effect to generate the photocurrent by breaking the momentum-inversion symmetry in the band structure. This mechanism induces a large photoconductivity in the AFM phase of the bilayer CrI_3_ despite no electric polarization. The photocurrent appears for the linearly polarized light but vanishes for the circularly polarized one in the 2D system. It exhibits an injection-current-like feature and is proportional to the relaxation time, the resonant transition rate and SOC. Tuning the magnetic structure is a sensitive handle to manipulate the photocurrent. Although the bilayer CrI_3_ exhibits a record photoconductivity, it seems possible to design even better nonlinear magnetic materials, for example by considering stronger SOC and longer relaxation time. There are plenty of AFM and FM materials, such as magnetoelectric multiferroic compounds^56,57^, waiting for exploration.

## Methods

We obtain the DFT band structure and Bloch wave functions from the Full-Potential Local-Orbital program (FPLO)^58^ within the generalized gradient approximation^59^. By projecting the Bloch wave functions onto atomic-like (Cr-*d* and I-*sp*) Wannier functions, we obtain a tight-binding Hamiltonian with sixty-eight bands that well reproduce the DFT band structure. We employ this material specific tight-binding Hamiltonian for accurate evaluation of the photocurrent. We use *ħ*/*τ* = 1 meV in our calculations. For the integrals of Eq. 1b, the 2D Brillouin zone was sampled by a grid of 400 × 400. Increasing the grid size to 960 × 960 varied the conductivity by less than 5%.The spin-orbit coupling was included in a self-conssistent way for the DFT calculations. The FPLO band structure is well consistent with other DFT methods.

## Supplementary information

Supplementary Information

Peer Review

## Data Availability

The data that support the findings of this study are available from the corresponding author upon request.
