# Supplementary material for: Switchable magnetic bulk photovoltaic effect in the two-dimensional magnet CrI3
Source: Nat Commun. 2019 Aug 22;10:3783. doi: 10.1038/s41467-019-11832-3 (PMC6706386; doi:10.1038/s41467-019-11832-3)
Supplement: Supplementary file 1 — Supplementary Information [file 41467_2019_11832_MOESM1_ESM.pdf]

Supplemental Materials for "Magnetically Switchable Light-Matter Interaction in the Two-Dimensional Magnet CrI_3"

Yang Zhang,[1,2,3] Tobias Holder,[4] Hiroaki Ishizuka,[5] Fernando de Juan,[6,7] Naoto Nagaosa,[8,9] Claudia Felser,[1] and Binghai Yan *[10]

[1]*Max Planck Institute for Chemical Physics of Solids, 01187 Dresden, Germany*

[2]*Leibniz Institute for Solid State and Materials Research, 01069 Dresden, Germany*

[3]*Department of Physics, Massachusetts Institute of Technology, Cambridge, Massachusetts 02139, USA*

[4]*Department of Condensed Matter Physics, Weizmann Institute of Science, Rehovot 7610001, Israel*

[5]*Department of Applied Physics, University of Tokyo, Tokyo 113-8656, Japan*

[6]*Donostia International Physics Center, P. Manuel de Lardizabal 4, 20018 Donostia-San Sebastian, Spain*

[7]*IKERBASQUE, Basque Foundation for Science, Maria Diaz de Haro 3, 48013 Bilbao, Spain*

[8]*RIKEN Center for Emergent Matter Science (CEMS), Wako 351-0198, Japan*

[9]*Department of Applied Physics and Quantum Phase Electronics Center (QPEC), University of Tokyo, Tokyo 113-8656, Japan*

[10]*Department of Condensed Matter Physics, Weizmann Institute of Science, Rehovot 7610001, Israel*

Email: binghai.yan@weizmann.ac.il

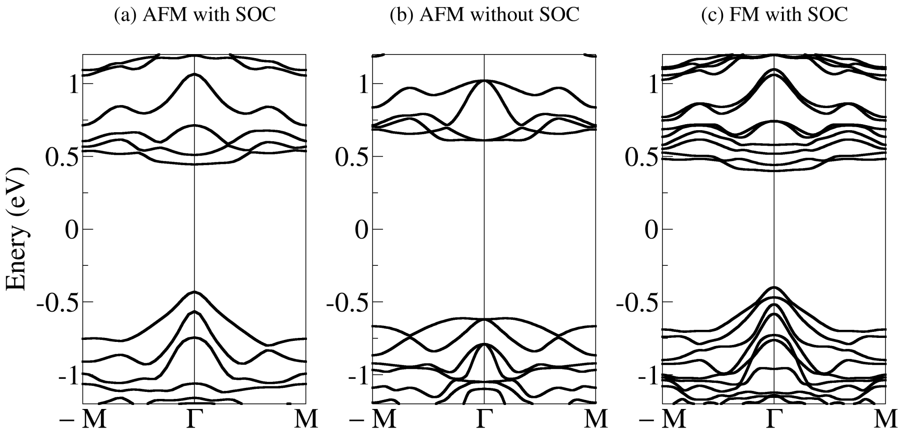

Supplementary Fig. 1. Band structure of the bilayer CrI_3 for the (a) AFM phase with SOC, (b) AFM without including SOC and (c) FM phase with SOC. In both (b) and (c), the $\mathbf{k}$ to $-\mathbf{k}$ symmetry is preserved in the band structure. In contrast, momentum inversion symmetry is broken in (a).

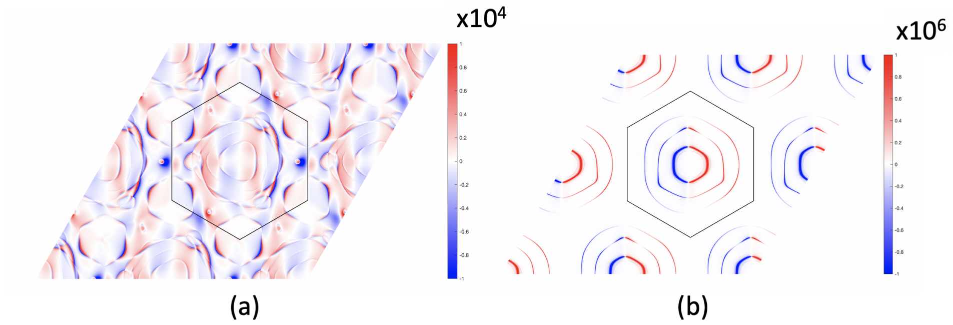

Supplementary Fig. 2. The photoconductivity contributions from (a) the three-band and (b) two-band processes. Distribution of the photoconductivity σ^x_{xx} ($\hbar\omega = 1.2$ eV) in the first Brillouin zone, the hexagonal area. Note that the colorbar of the three-band contribution is two orders in magnitude smaller than the two-band one. The momentum inversion symmetry is broken in both (a) and (b). The three-band distribution is relatively uniform in the Brillouin zone, because it has no energy selection rule.

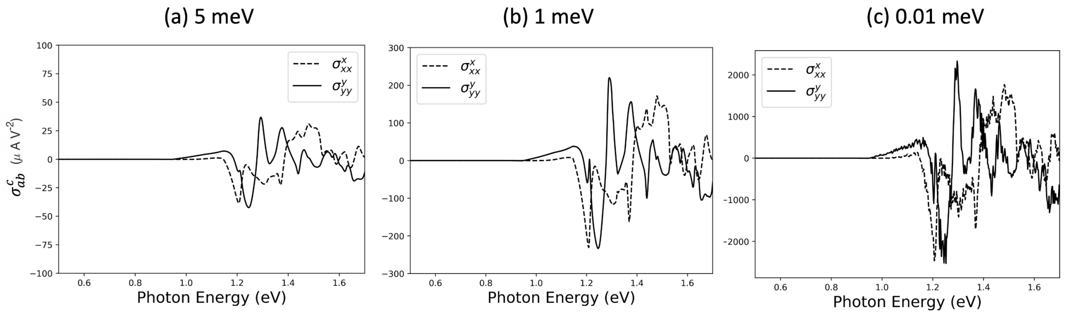

Supplementary Fig. 3. The photoconductivity for different relaxation time (a) $\hbar/\tau = 5$ meV, (b) 1 meV (same as Fig. 2a) and (c) 0.01 meV. The photoconductivity scales linearly with τ.

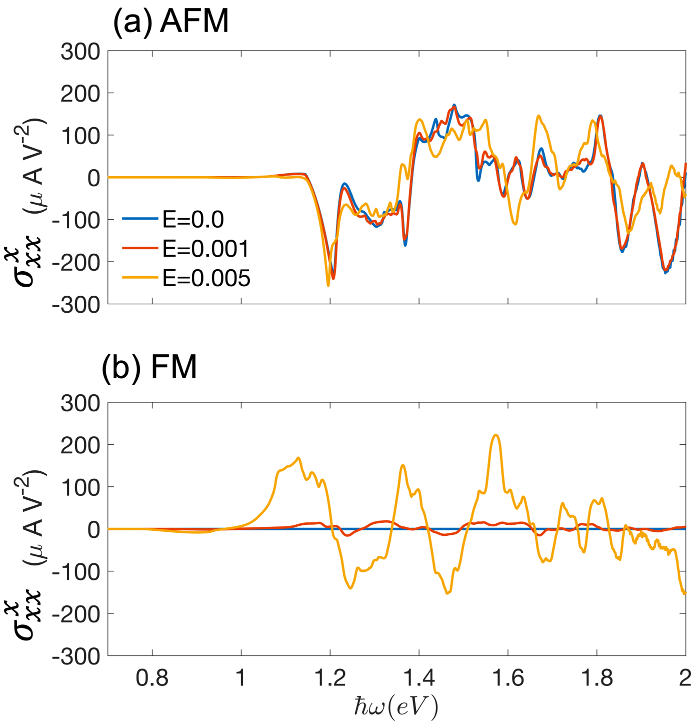

Supplementary Fig. 4. The photoconductivity σ^x_{xx} in different electric field applied for the (a) AFM and (b) FM phases. The electric field E is in unit of V/$a.u.$ (1 $a.u.$ = 0.53 Å).
