# Supplementary material for: Switchable magnetic bulk photovoltaic effect in the two-dimensional magnet CrI3
Source: Nat Commun. 2019 Aug 22;10:3783. doi: 10.1038/s41467-019-11832-3 (PMC6706386; doi:10.1038/s41467-019-11832-3)
Supplement: Supplementary file 2 — Peer Review [file 41467_2019_11832_MOESM2_ESM.pdf]

Reviewers' comments:

Reviewer #1 (Remarks to the Author):

The authors present a calculation of the 2nd order DC current present in insulating antiferromagnet which is invariant under combined operations of parity (P) and time reversal (T), but which is not invariant under P and T individually. The material exhibits a unique response which has features typically associated with materials that break P - e.g. a "shift current" response leading to DC current due to excitation from linearly polarized light - combined with features associated with "injection current", namely a response that varies linearly with momentum scattering time \tau.

My view of the results is that they are not entirely surprising, although as far as I'm aware, the 2nd order response of this type of system has not been considered before. However I think the paper is well executed, and the results exhibit some features that are unique from a formal point of view (e.g. a shift current that's linear with \tau), and potentially useful in the context of antiferromagnets and optical spintronics. It is therefore my opinion that the work merits publication in Nature Communications.

One small comment: to provide the reader with more detailed understanding of the system, some commentary on the shapes of the response in Fig. 2(c),(d) would be helpful. For example, why are the \sigma^x maxima shifted away from \theta=0,180?

Reviewer #2 (Remarks to the Author):

In their manuscript, Zhang et.al. proposed a magnetic photo-galvanic effect (MPGE) in bilayer CrI3. The key finding presented in manuscript is based on "k and –k symmetry breaking" in the band structure of the AFM phase shown in Fig. 1(d). Such a symmetry broken is surprising. I notice that the calculations are done within a Full-Potential Local-Orbital program (FPLO)[53]. Could the authors reproduce Fig.1(d) within VASP or Wien2k code? How about the calculation results with charge self-consistence in the presence of spin-orbit coupling?

Reviewer #3 (Remarks to the Author):

The authors have report the novel magnetic bulk photovoltaic effect (MPGE) because of the spin-orbit coupling breaking SU(2) symmetry for the bilayer two dimensional AFM CrI3. Based on the first-principles calculation, the authors demonstrate the MPGE is much larger than any previously reported results from other mechanisms including the shift current. These findings are important and interesting for the potentially devices combining magnetic, electronic, and optical functionalities. So, I suggest the paper to be considered as an article unless a list of questions that I think need to be addressed.

(1) One of the major finds, i.e. MPGE is much larger than any previously reported results from other mechanisms including the shift current, lies on the large relaxation time approximation. For example, the authors get more than 200 μAV^{-2} current for a relaxation time $\tau \approx 0.4$ ps. However, the relaxation time used in this paper is one-order larger than that of typical 2D materials such as MoS_2 (Physical Review Materials, 2, 114010(2018)). Since there is no calculation or experiment evidence about the long relaxation time of the carriers of CrI3, the authors should carefully give the statement about the MPGE current is much larger than other current includes shift current. In addition, does the relaxation time relate to the spin-relaxation time because this MPGE is induced by the spin-orbit coupling. The authors should give appropriate comments.

(2) The authors discuss the difference between shift current and MPGE using equation 1. It is clear that the Eq.1 can describe the injection current and MPGE, but it is not clear for me that this equation can also describe the shift current which does not have relaxation-time. The authors should describe the shift current using Eq. 1 more clearly.

(3) All the equations in this manuscript seem to miss 1/hbar factor. This factor is important to give correct units and magnitude of MPGE. The authors should carefully check the equations with their first-principle calculation.

(4) The authors discuss that the numerator N_lmn(k) is real for this PT symmetrical material and get the real part of current by the Eq.4 or Eq. 5. However, the imaginary part in MPGE cannot be cancelled, what is the physical meaning of imaginary part in this MPGE DC current? As we know, the imaginary part is cancelled for injection current or shift current.

In addition to these, here are some small comments

(5) The exciton effect is important, particularly for these 2D materials. The authors should comment the exciton effect on MPGE, and cite the relevant references (arXiv:1811.05287, and arXiv:1904.12813).

(6) The authors discuss the AFM and FM states of bilayer CrI3, the relevant experiment references should be cited (Nature Physics, 14(3), 277(2018)).

======================Reply to Referees' comments==============

---------------Reviewer #1 (Remarks to the Author):--------------------

The authors present a calculation of the 2nd order DC current present in insulating antiferromagnet which is invariant under combined operations of parity (P) and time reversal (T), but which is not invariant under P and T individually. The material exhibits a unique response which has features typically associated with materials that break P - e.g. a "shift current" response leading to DC current due to excitation from linearly polarized light - combined with features associated with "injection current", namely a response that varies linearly with momentum scattering time \tau.

My view of the results is that they are not entirely surprising, although as far as I'm aware, the 2nd order response of this type of system has not been considered before. However I think the paper is well executed, and the results exhibit some features that are unique from a formal point of view (e.g. a shift current that's linear with \tau), and potentially useful in the context of antiferromagnets and optical spintronics. It is therefore my opinion that the work merits publication in Nature Communications.

One small comment: to provide the reader with more detailed understanding of the system, some commentary on the shapes of the response in Fig. 2(c),(d) would be helpful. For example, why are the \sigma^x maxima shifted away from \theta=0,180?

Reply: We thank the referee for his/her positive comments and recommendation of publication.

Here we add the further explanation on Fig 2 (c) (d). Following Eq.2, we point out that \sigma^x is dependent on \sigma^x_xx and \sigma^y_yy. If sigma^y_yy is zero, the maxima of \sigma^x is located at θ=0,180 degree. Because \sigma^yyy is generically nonzero at a given frequency, the maxima of \sigma^x shift away from θ=0,180 degree. We have added related explanation in the text.

------------------Reviewer #2 (Remarks to the Author):----------------------

In their manuscript, Zhang et.al. proposed a magnetic photo-galvanic effect (MPGE) in bilayer CrI3. The key finding presented in manuscript is based on "k and –k symmetry breaking" in the band structure of the AFM phase shown in Fig. 1(d). Such a symmetry broken is surprising. I notice that the calculations are done within a Full-Potential Local-Orbital program (FPLO)[53]. Could the authors reproduce Fig.1(d) within VASP or Wien2k code? How about the calculation results with charge self-consistence in the presence of spin-orbit coupling?

Reply:

We thank the referee for the comment on our computational method. And we have

reproduced the Fig(1d) with VASP as suggested, in the following figure. One can also find the same surprising k to -k symmetry breaking.

In addition, the charge self-consistence is carried out in the presence of spin-orbit coupling both in VASP and FPLO.

We have added corresponding clarification in the supplementary information.

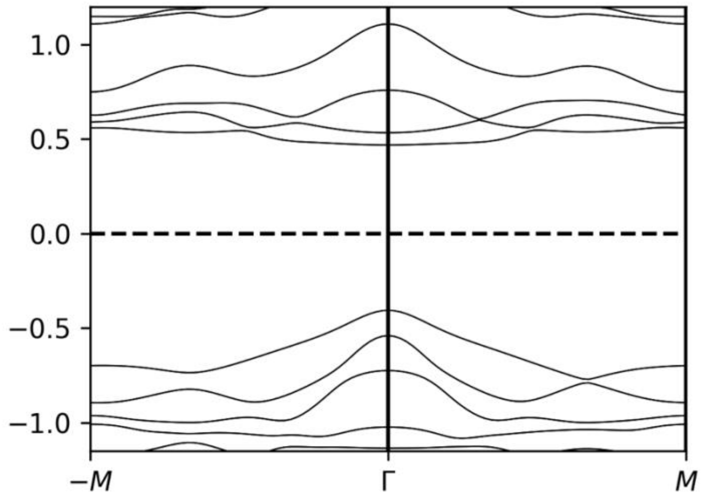

Figure 1. Band structure from VASP

----------------Reviewer #3 (Remarks to the Author):-----------------------------

The authors have report the novel magnetic bulk photovoltaic effect (MPGE) because of the spin-orbit coupling breaking SU(2) symmetry for the bilayer two dimensional AFM CrI3. Based on the first-principles calculation, the authors demonstrate the MPGE is much larger than any previously reported results from other mechanisms including the shift current. These findings are important and interesting for the potentially devices combining magnetic, electronic, and optical functionalities. So, I suggest the paper to be considered as an article unless a list of questions that I think need to be addressed.

Reply: We thank the referee for his/her comments on our work. We have addressed these questions in the following.

(1) One of the major finds, i.e. MPGE is much larger than any previously reported results from other mechanisms including the shift current, lies on the large relaxation time approximation. For example, the authors get more than 200 μAV−2 current for a relaxation time τ ≈ 0.4 ps. However, the relaxation time used in this paper is one-order larger than that of typical 2D materials such as MoS2 (Physical Review

Materials, 2, 114010(2018)). Since there is no calculation or experiment evidence about the long relaxation time of the carriers of CrI3, the authors should carefully give the statement about the MPGE current is much larger than other current includes shift current. In addition, does the relaxation time relate to the spin-relaxation time because this MPGE is induced by the spin-orbit coupling. The authors should give appropriate comments.

Reply: This is an expert question. The key feature of MPGE proposed is that the photocurrent is proportional to the inter-band relaxation time. In the Supplementary Table 1 of this experiment paper [Nature Communications 5: 4543 (2014)] https://media.nature.com/original/nature-assets/ncomms/2014/140729/ncomms5543/extref/ncomms5543-s1.pdf different types of relaxation time for MX2 are well summarized to overview the literature. Relevant carrier lifetime (except the spin lifetime ~ns) is in the range of 0.3 to 100 ps. Because there is no experiment report on the relaxation time for CrI3, we chose a value of $\tau \approx 0.4$ ps ($\hbar/\tau = 1$ meV), which is near the lower boundary of MX2. We also added the photocurrent results for larger and shorter τ in the supplementary Figure S3. To avoid overclaiming, we rephrased the statement in the text by stressing the relaxation time approximation. In addition, we cited the above paper and the theory paper mentioned by the referee.
Because the essential point to generate MPGE is the carrier velocity imbalance, which is irrelevant to the spin polarization, the relaxation time here is not related to the spin-relaxation time.

(2) The authors discuss the difference between shift current and MPGE using equation 1. It is clear that the Eq.1 can describe the injection current and MPGE, but it is not clear for me that this equation can also describe the shift current which does not have relaxation-time. The authors should describe the shift current using Eq. 1 more clearly.
Reply: Historically the shift current formalism was actually derived from Eq. 1 (Ref.2). Our Eq. 1b was reformulated from the Eq. (7) in Ref.2.

$$\vec{j} = I_0 \frac{|e|^3}{4\pi^3 m_0^3 \epsilon_0 c \eta \omega^2} \mathrm{Re} \sum_{\Omega=\pm\omega} \sum_{l,m,n} \int_{\text{first BZ}} d^3k (f_l - f_n) \frac{\langle n,\vec{k}|\vec{e}\cdot\vec{p}|l,\vec{k}\rangle\langle l,\vec{k}|\vec{e}\cdot\vec{p}|m,\vec{k}\rangle\langle m,\vec{k}|\vec{p}|n,\vec{k}\rangle}{(E_n - E_m - i\delta)(E_n - E_l + \hbar\Omega - i\delta)} . \qquad (7)$$

By assuming a long relaxation-time, the shift current was obtained in Eq. (19) in Ref.2. We point that the shift vector in its Eq.(20) is nearly the same formalism as the Berry connection, which is more familiar today.

$$\vec{j} = I_0 \frac{|e|^3}{4\pi^2 m_0^2 \epsilon_0 c \hbar \eta \omega^2} \sum_{l,n} \int_{\text{first BZ}} d^3k (f_n - f_l) \delta(E_n - E_l - \hbar\omega) \,\mathrm{Im}[\langle n,\vec{k}|\vec{e}\cdot\vec{p}|l,\vec{k}\rangle\langle l,\vec{k}|(\vec{e}\cdot\vec{p})\vec{R}_{n,\vec{k}} + \vec{R}^*_{l,\vec{k}}(\vec{e}\cdot\vec{p})|n,\vec{k}\rangle] . \qquad (19)$$

The objects

$$\vec{R}_{\rho,\vec{k}}(\vec{r}) = \frac{\nabla_{\vec{k}} u_{\rho,\vec{k}}(\vec{r})}{u_{\rho,\vec{k}}(\vec{r})} - \int_{\text{unit cell}} u^*_{\rho,\vec{k}}(\vec{r}') \nabla_{\vec{k}} u_{\rho,\vec{k}}(\vec{r}') d^3r' \qquad (20)$$

Because of the long-relaxation-time approximation, the denominator of Eq.1b can be reformulated into some delta-function as shown in Eq. (8) of Ref.2.

$$D_1 = \frac{1}{E_n - E_m - i\delta} = \frac{P}{E_n - E_m} + i\pi\delta(E_n - E_m)\,,$$
$$D_2 = \frac{1}{E_n - E_l + \hbar\Omega - i\delta} = \frac{P}{E_n - E_l + \hbar\Omega} + i\pi\delta(E_n - E_l + \hbar\Omega)\,, \quad (8)$$

This is the reason why the shift current formula does not include relaxation time.

(3) All the equations in this manuscript seem to miss 1/hbar factor. This factor is important to give correct units and magnitude of MPGE. The authors should carefully check the equations with their first-principle calculation.

Reply: It is insightful to judge a quantum mechanics effect by observing $\hbar$. Our equations (like Eq.1) indeed include $\hbar$, in the velocity operator 1/hbar dH/dk. The photo frequency is presented without hbar, we would get an additional 1/hbar in the prefactor as the referee recognized. Here we checked the unit and the equation gives the correct unit for photocurrent,

(4) The authors discuss that the numerator N_lmn(k) is real for this PT symmetrical material and get the real part of current by the Eq.4 or Eq. 5. However, the imaginary part in MPGE cannot be cancelled, what is the physical meaning of imaginary part in this MPGE DC current? As we know, the imaginary part is cancelled for injection current or shift current.

Reply: Since the observed photocurrent $\mathrm{J}^{\mathrm{c}} = \sum_{\mathrm{ab}} \sigma_{ab}^{\mathrm{c}}\, \mathrm{E}_{\mathrm{a}}^{*}(\omega)\mathrm{E}_{\mathrm{b}}(\omega)$ is always real, it requires σ_{ab}^{c} to be real for a linearly polarized light. However, σ_{ab}^{c} should be imaginary for the circularly polarized light, because $\mathrm{E}_{\mathrm{a}}^{*}(\omega)\mathrm{E}_{\mathrm{b}}(\omega)$ is purely imaginary then. Because the circular photogalvanic effect vanishes for CrI3, as we mentioned in the middle of Page 8, we do not focus on it.

In addition to these, here are some small comments

(5) The exciton effect is important, particularly for these 2D materials. The authors should comment the exciton effect on MPGE, and cite the relevant references (arXiv:1811.05287, and arXiv:1904.12813).

(6) The authors discuss the AFM and FM states of bilayer CrI3, the relevant experiment references should be cited (Nature Physics, 14(3), 277(2018)).

Reply: Thanks for pointing out the important references. We have cited the suggested works in the revised manuscript, as Refs. 40, 45 & 46.

REVIEWERS' COMMENTS:

Reviewer #2 (Remarks to the Author):

In their manuscript, the authors perform first-principles calculations and find a magnetism-induced asymmetry of the carrier velocity in band structure of bilayer CrI3, and then propose a general scheme of magnetic bulk photovoltaic effect.

I have read the revised manuscript as well as the response letter. The manuscript is well written, the finding presented in the manuscript is interesting, and the analysis is convincing. I therefore recommend it publication on Nat. Comm. without any reservation.

Reviewer #3 (Remarks to the Author):

The author addressed most of my questions, so I suggest it be published.
